# COVID-19 neurological manifestations: correlation of cerebral MRI imaging and lung imaging—Observational study

**DOI:** 10.1186/s43055-021-00630-x

**Published:** 2021-10-04

**Authors:** Rasha Aly Saleh, Ekhlas Shaban

**Affiliations:** Faculty of Medicine, Tanta University, Tanta, Egypt

## Abstract

**Background:**

During the current pandemic, there is an increased incidence of neurologic/neuropsychiatric manifestations in patients with the novel coronavirus (COVID-19). Neurologic manifestations may be coincident or result of disease and its therapy. In the emergency department, orientation of the clinician with this issue is crucial for accurate decision making to limit the spread of infection during neurologic treatment. This study aimed to be familiar with MRI findings in patients with Neuro-COVID. Seventy patients presented with neurologic/neuropsychiatric manifestation either post COVID or during hospitalization underwent cerebral MRI from April 2020 to June 2021 (39 men and 31women; mean age 43.27, age range from 16 to 69 years).

**Results:**

Headache (80%), is the most prevalent neurological manifestations followed by smell and taste impairment (62.9%) and stroke symptoms (45.7%). Low mood and anxiety (17.1%), prolonged fatigue (14.3%) and depression (7.1%) are the most common psychiatric symptoms. Infarctions, hematoma and demyelinating disease are the most prevalent findings. There is a week positive correlation between MRI findings and CT chest finding but without statistical significance (*P*-value 0.2).

**Conclusions:**

Cerebrovascular disease and demyelinating lesions are common manifestations of COVID 19. Familiarity of neurologists and radiologist in the emergency department and in-patient with this issue is crucial to avoid misdiagnosis and the spread of infection.

## Background

The coronaviruses (CoVs) are a large group of positive-strand RNA viruses characterized by club-like spikes that project from their surface, an unusually large RNA genome, and a unique replication strategy. CoVs cause various diseases range from mild respiratory disease, enteritis and potentially lethal respiratory infections [1].

In March 2020, the World Health Organization (WHO) declares 2019 novel coronavirus (COVID 19) that was first identified in Wuhan, China in December 2019 as a global pandemic. By June 2021, There have been 175,541,600 confirmed cases of COVID-19, including 3,798,361 deaths, reported to WHO [2].

COVID-19 is highly contagious disease. Although the respiratory system remains the most primarily affected organ, a wide-ranging multi-organ disease has evolved. Several case reports revealed that the neurotropic and neuroinvasive properties of the virus leading to neurological diseases [3].

Neurological symptoms in COVID-19 patients may arise due to (1) Viremia with subsequent injury to vascular endothelial and epithelial cells (disruption of BBB), (2) hypoxic injury due to respiratory failure and prolonged ventilation that facilitated entry of the virus into neural tissue cells, (3) retrograde -upper nasal trans-cribrial neuronal route and direct damage to the olfactory and gustatory receptors (neurotropism of CoVs enables the COVID-19 to reach the brain), (4) immune injury secondary to cytokine storm syndrome and heighten state of inflammation, (5) COVID-19 induced coagulopathy, (6) COVID-19 bind and engage with the ACE2 receptors, COVID-19 docks on the ACE2 via spike protein (lungs, heart, kidneys, intestines, brain, and testicles are well-known to express ACE2 receptors) and are possible targets of COVID-19 [4].

Aim: To be familiar with MRI findings in patients with Neuro-COVID.

## Methods

### Patients

A single-center prospective observational study was conducted at a tertiary care center in a University Hospital. Seventy patients with past or present history of typical COVID-19 infection who presented with neurologic/ neuropsychiatric symptoms (with or without respiratory symptoms) between April 2020 and June 2021. Demographic data (age, sex), medical history of comorbid diseases (diabetes mellitus, hypertension, chronic kidney disease, cardiac disease, cancer, SLE, RA), complete clinical and neurological examination and laboratory findings were done.

Patients with previous neurocognitive disorders were excluded from the study. An ethical approval was obtained from local ethics committee.

Neuro-COVID manifestations were categorized into two classes: I. neurologic manifestations including headache, impaired consciousness, and acute cerebrovascular disease, extrapyramidal manifestation, anosmia, ageusia, II. psychiatric symptoms including low mood and anxiety, depression, prolonged fatigue and insomnia.

### Method

- MRI using a 1.5 T MR scanner (Signa;16 channel, Excite, GE Healthcare, Milwaukee, WI, USA). MR sequences parameters were as follows: T1-weighted images parameters: (TR:360 ms, TE: 9 ms), T2-weighted images parameters (TR:6672 ms, TE: 147 ms), GRE T2* (TR: 46.5, TE: 2.5, flip angle: 10°). FLAIR (TR: 7432, TE: 118.6, inversion time (TI):2200). Diffusion weighted images with B value 1000. Examination with a head coil. Field of view (FOV) 22 × 18 mm; matrix, 310 × 620; slice thickness, 3 mm and slice gap, 0.4 mm. MRV (TR: 20.1, TE: 5.2, Flip angle: 32, Matrix 235, FOV:200).
- CT chest at time of admission for patient in acute phase and new CT chest for patient with post-COVID new symptoms to rule out possibility of re-infection.
- Image interpretation and analysis:

Two radiologists interpreted the images in conjunction. One reader (E.S) had 14 years of experience interpreting, and the second reader (R.S) had 15 years of experience.

### Statistics

Data were described as number, mean ± standard deviation (SD) and frequency (percent) for categorical data. The distribution of categorical data was assessed using chi square test (with exact *p*-value). *P* < 0.05 was considered as significance level. Correlation of finding were tested using Pearson and Spearmen test. All statistical analyses were analysed using IBM SPSS software package version 25.0 (Armonk, NY: I BM Corp).

## Results

Seventy patients presented neurologic/ neuropsychiatric symptoms with typical COVID-19 infection; age ranged from 16 to 69 years with the mean age of 43.27 ± 13.99 years were enrolled. (55.7% male, 44.3 female). Post-COVID-19 neuro-COVID was detected in 62 out of 70 patient (88.6%), while 8 out of 70 patient (11.4%) developed neurological symptoms during admission with typical COVID. The most prevalent neurological manifestations in the studied participants. Headache (80%), and smell and taste impairment (62.9%), followed by stroke symptoms (45.7%) were the most prevalent neurologic symptoms. Low mood and anxiety (17.1%) were the most common psychiatric symptoms, followed by prolonged fatigue (14.3%). Depression was the least common neuropsychiatric symptoms represented (7.1%) (Table 1).

**Table 1:** The frequency of neurological manifestations in patients with COVID-19

	Variable	Number	percent
Age (16–69 years)	Mean	43.27	
	Std. deviation	13.998	
Gender	Male	39	55.7
	Female	31	44.3
Neurologic symptoms*	Headache	56	80
	Anosmia/hypogeusia	44	62.9
	Stroke symptoms	32	45.7
	Disturbed consciousness	20	28.6
	Seizers	5	7.1
	Extra-pyramidal (Parkinson like)	1	1.4
Neuropsychiatric symptoms	No neuropsychiatric symptoms	43	61.4
	Low mood and anxiety	12	17.1
	Prolonged fatigue	10	14.3
	Depression	5	7.1
Serious non neurologic association	C/P: Edema around eye	1	1.4
	MRI: Fungal sinusitis		
Outcome	Survivors	47	67.1
	Death	12	17.1
	Unclear	11	15.7

*Multiple patients had multiple symptoms

The majority (62.9%) had comorbidities. The most common comorbidity was HTN and diabetes (Table 2).

**Table 2:** The frequency of comorbidity and neurological manifestations in patients with COVID-19

Comorbidity	Frequency	Percent	Valid Percent
No comorbidity	26	37.1	37.1
*Single comorbidity*			
HTN	11	15.7	15.7
SLE	4	5.7	5.7
DM	4	5.7	5.7
Cardiac	4	5.7	5.7
CKD	3	4.3	4.3
Morbid obesity	3	4.3	4.3
Rheumatoid arthritis	1	1.4	1.4
Lymphoma	1	1.4	1.4
*Multiple comorbidity*			
DM and HTN	4	5.7	5.7
Obesity and DM	3	4.3	4.3
Cardiac and HTN	1	1.4	1.4
Obesity and HTN	1	1.4	1.4
Lymphoma and HTN	1	1.4	1.4
Obesity, HTN, Cardiac	1	1.4	1.4
SLE, DM, HTN	1	1.4	1.4
Total	70	100.0	100.0

Total HTN patients were 20 (28.6%), Total DM patients were 12 (17.1%), total morbid obesity patient 8 (11.4%), total cardiac patient 6 (8.6%), total lymphoma patients were 2 (2.9%)

Lung imaging, revealed that CO-RAD IV and V (75.7%), were the most common CT findings followed by CO-RAD III (20%) (Table 3).

**Table 3:** The frequency of chest findings in examined patients

CT chest	Frequency	Percent
CO-RAD I	2	2.9
CO-RAD II	1	1.4
CO-RAD III	14	20.0
CO-RAD IV	27	38.6
CO-RAD V	26	37.1
Total	70	100.0

**Fig. 1:**
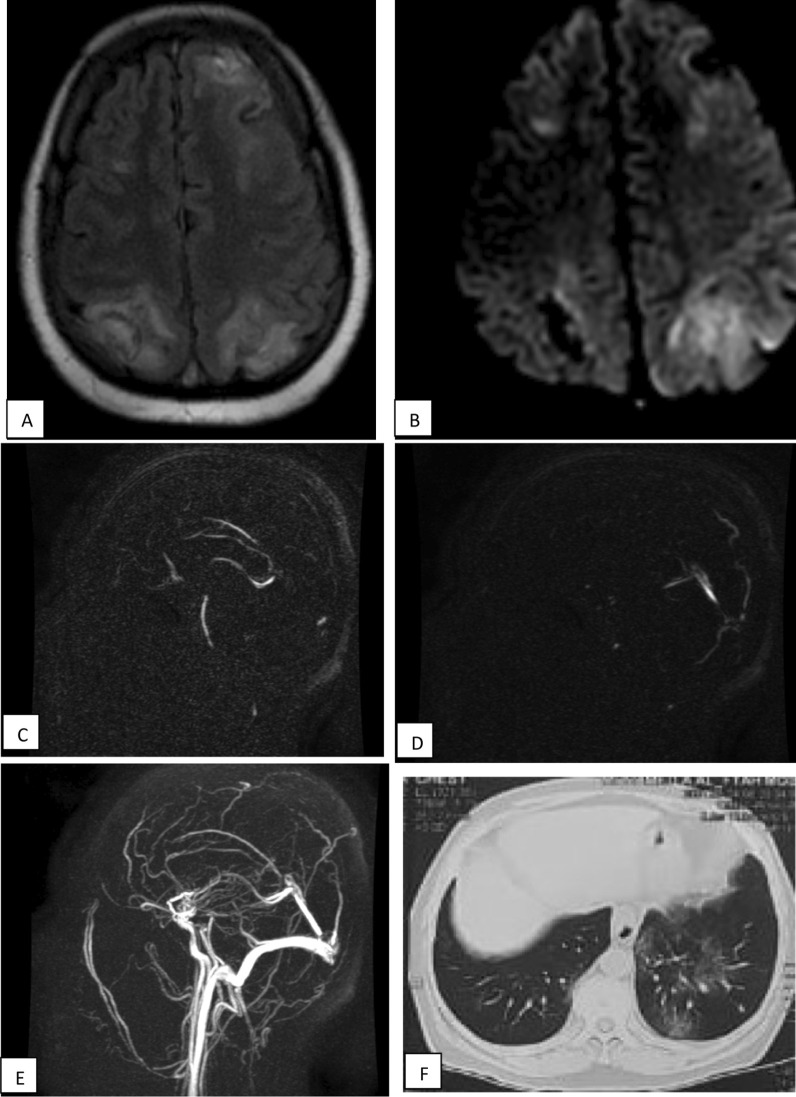
A 36-year-old male patient, presented with post-COVID headache, seizers and disturbed consciousness. **a** MRI axial T2WI revealed cortical and subcortical regions of high T2-signal intensities represents non haemorrhagic venous infarctions. **b** Corresponding regions of restricted diffusion in DWI with b-value1000. **c**, **d** and **e** MRV show near complete obstruction of the superior sagittal sinus. Non haemorrhagic venous infarction is confirmed. **f** CT chest showed peripheral distributed ground glass opacities typical for COVID-19 (CORAD-V)

**Fig. 2:**
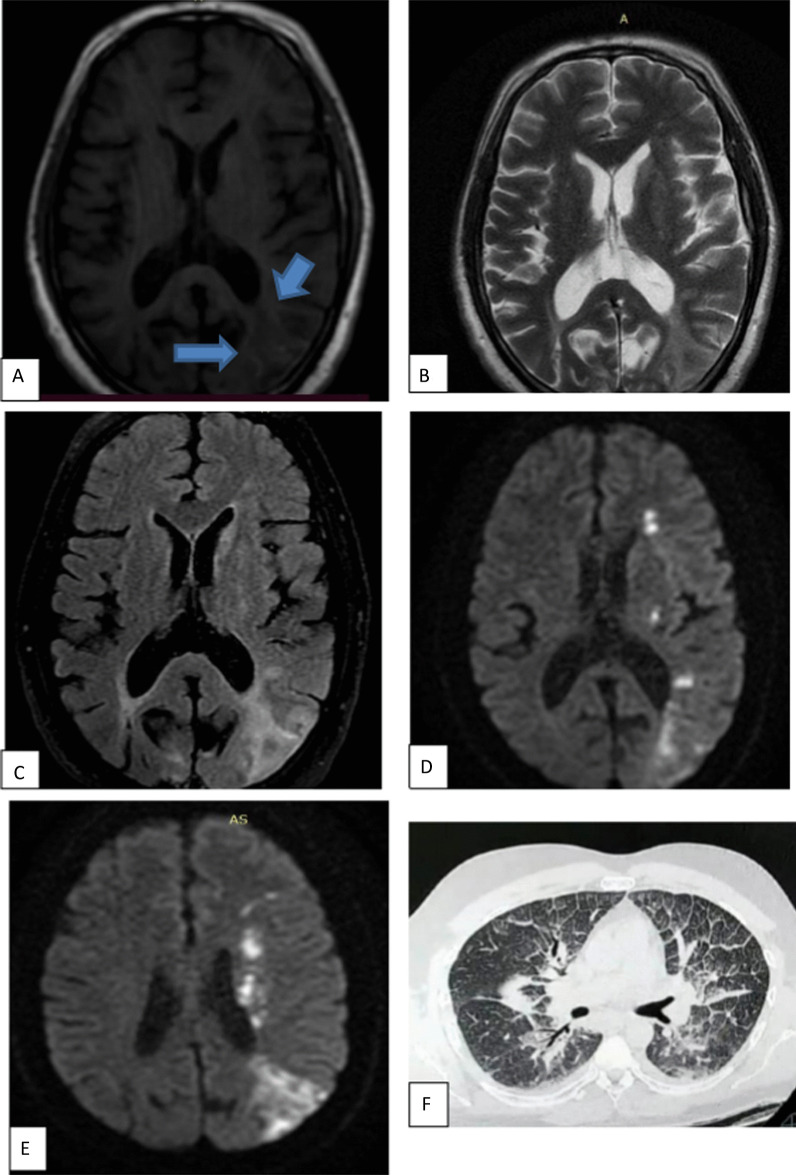
A 43-year-old female patient presented with post COVID fatigue, followed by cerebrovascular stroke symptoms. MRI axial T1WI (**a**) revealed left occipital subcortical white matter abnormal signal display relative low signal intensity (arrow). Axial T2WI (**b**) show high SI with no mass effect. Axial FLAIR (**c**) showed corresponding increase signal intensity. DWI (**d**, **e**) showed areas of restricted diffusion. watershed infarcts with additional foci of lacunar infarcts. CT lung showed GGO very typical for COVID (CORAD-V)

**Fig. 3:**
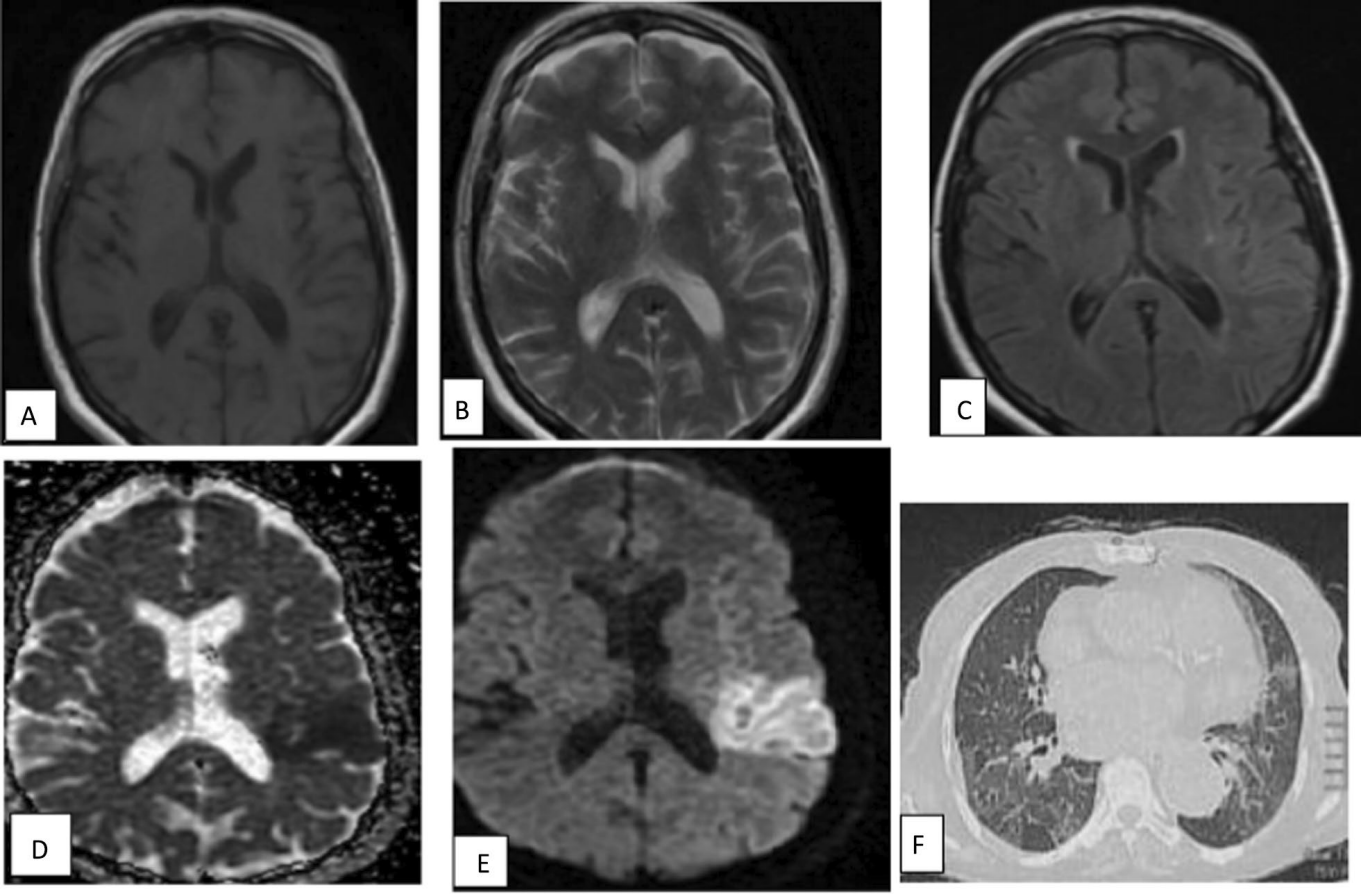
A 50-year-old male patient presented with cerebrovascular stroke post COVID. MRI **a** axial T1WI, **b** axial T2WI, **c** axial FLAIR revealed no abnormality. **d** ADC map, **e** DWI revealed a well-defined area of restricted left parietal region acute infarction. **f** CT chest at admission showing CORAD III (mosaic CT attenuation). CT chest during acute phase is not available

**Fig. 4:**
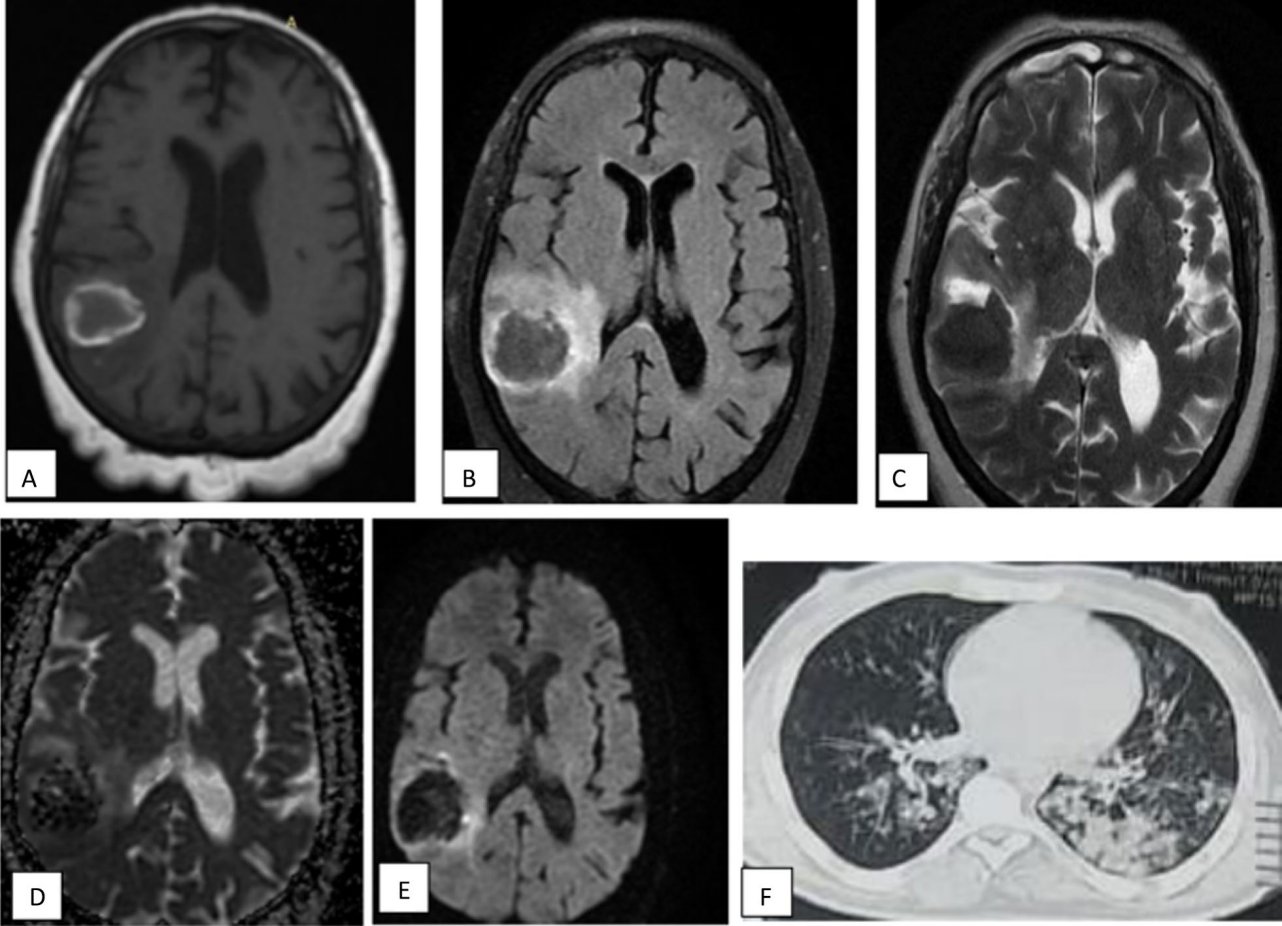
A 50-year-old male presented with sever continuous headache during COVID infection, expressive aphasia. MRI revealed right occipital well defined intra axial space occupying lesion. **a** Axial T1 WI showed area of peripheral high SI with central area of iso intense signal intensity. **b** Axial FLAIR show area of peripheral edema (**c**) axial T2WI showed area of relative low SI with peripheral edema (**d**) ADC map and (**e**) DWI show area of peripheral restriction. Finding correlates with subacute intraparenchymal hematoma. **f** CT lung showed multifocal GGO and consolidation, very typical COVID abnormalities (CORAD-V)

**Fig. 5:**
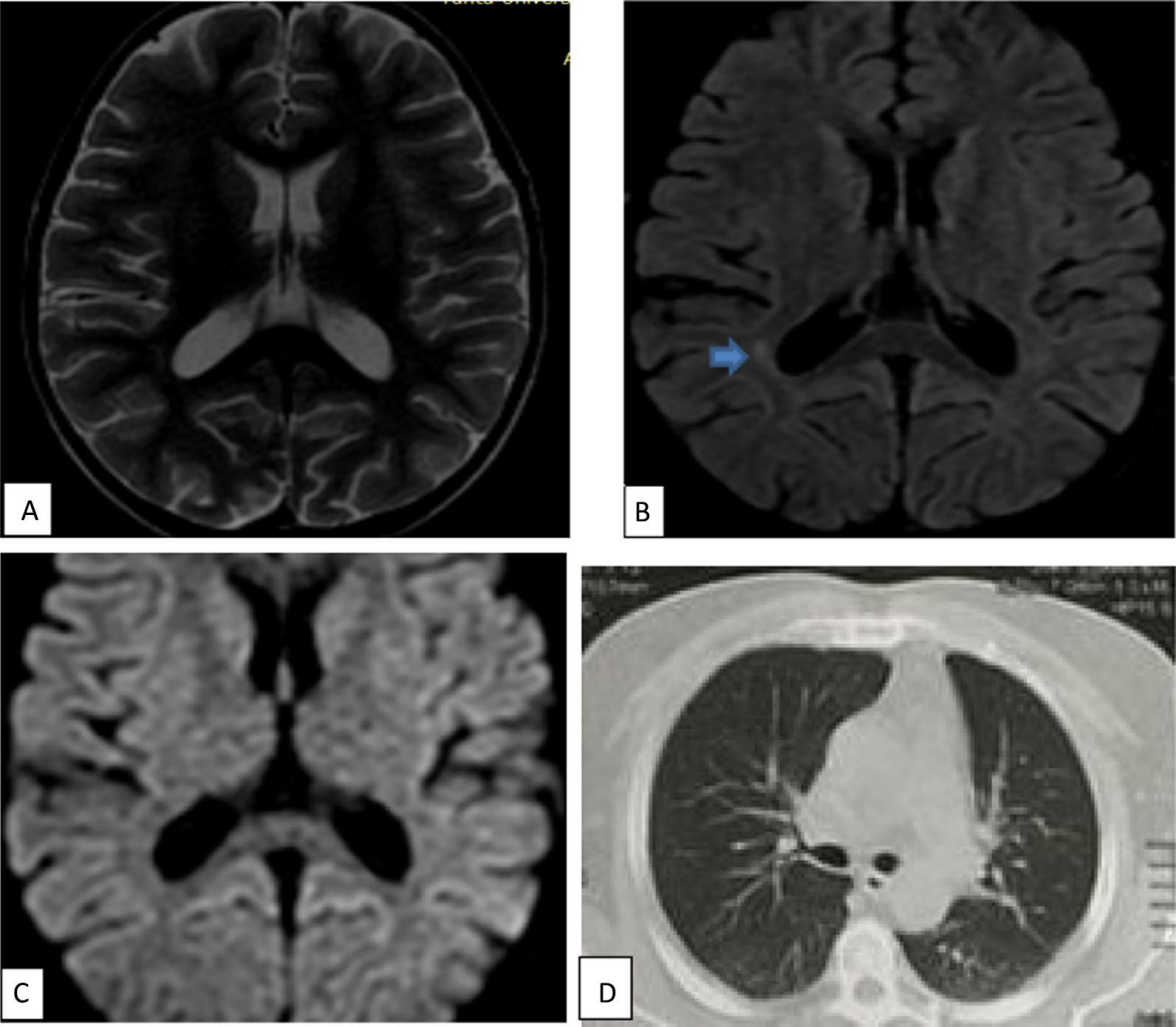
A 17-year-old female patient with chronic kidney disease presented with post COVID puerperal skin rash, headache and decreased conscious level. MRI axial T2WI (**a**) hardly detect abnormal signal and axial FLAIR (**b**) revealed abnormal foci of increased signal intensity in right periventricular deep white matter (blue arrows). Diffusion images (**c**) show no restricted diffusion. Post COVID vasculitis was considered. CT chest during acute phase not shown. CT chest at time of admission show no abnormalities (CORAD-I)

**Fig. 6:**
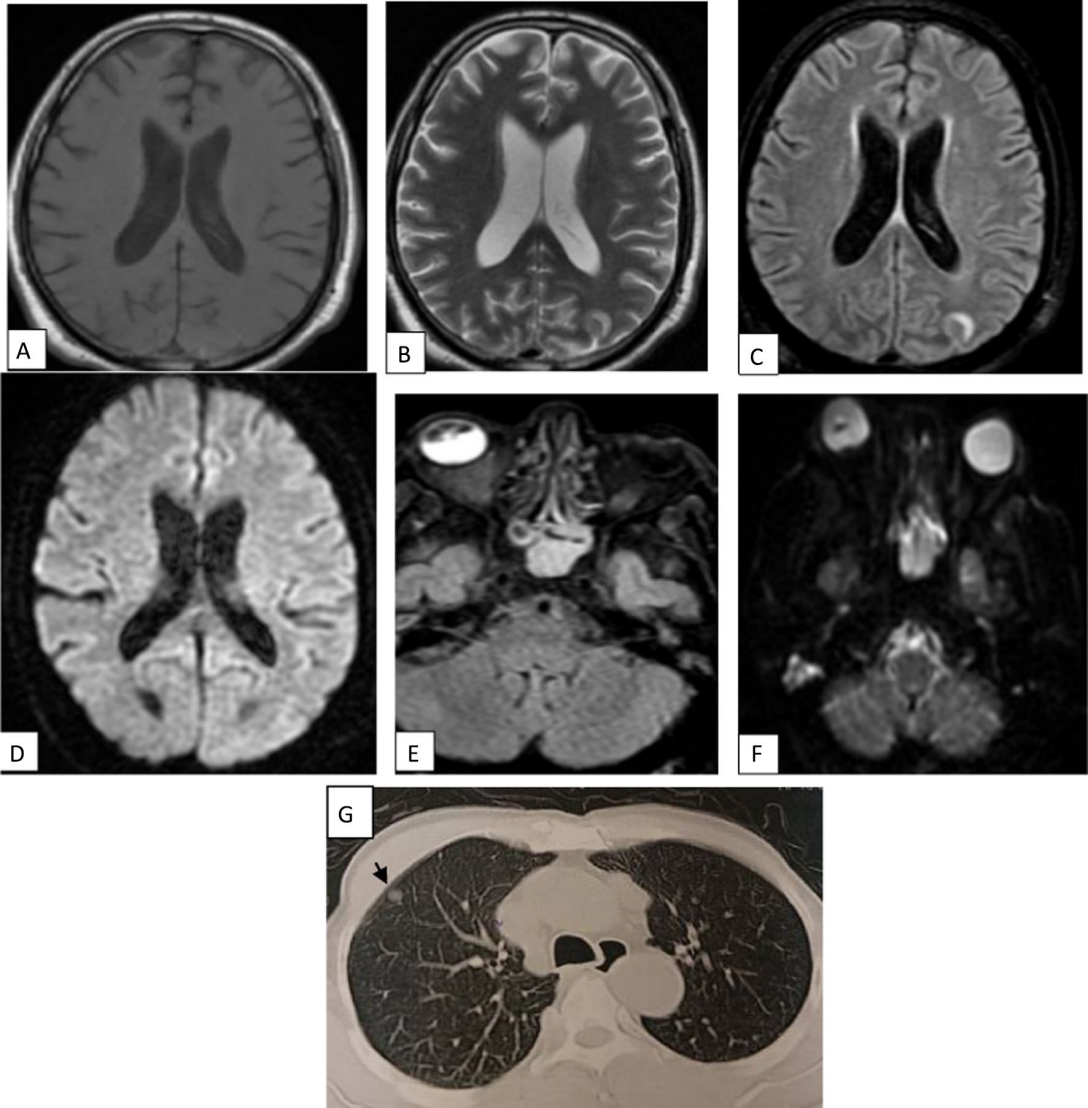
A 47-year-old male patient presented with post COVID ophthalmoplegia in six cardinal directions, MRI **a** axial T1WI, **b** axial T2& **c** axial FLAIR **d** DWI show left occipital gyral edema with no diffusion restriction. In addition to ethmoidal sinusitis which show restricted diffusion in DWI (**e**) and of high FLAIR SI (**f**), histopathologically proved to be invasive fungal sinusitis. CT chest showed pulmonary nodule (short black arrow) probably post COVID infection sequel CORAD-III

The most common MRI finding were cerebrovascular stroke: infarction 37.14% (Figs. 1, 2 and 3), hematoma 20% (Fig. 4), vasculitis 15.7% (Fig. 5), demyelinating disease 14.28% (ADEM 7.1%, PRES 7.1%), gyral edema 5.7% (Fig. 6) and common WM micro haemorrhage 2%) (Table 4).

**Table 4:** The MRI findings of neurological manifestations in patients with COVID-19

MRI findings		N	Percent	N/(Percent)
Infarction	Acute large infarction	9	12.9	26 (37.14%)
	Subacute large infarction	4	5.7	
	Lacunar infarction	7	10	
	Non Hgic Venous infarction	3	4.3	
	Hemorrhagic venous infarction	3	4.3	
Intracerebral hemorrhage	Hematoma	14	20	16 22.9%
	WM micro-hemorrhage	2	2.9	
Vasculitis	Vasculitis	11	15.7	11 15.7
Demyelinating disease	ADEM	5	7.1	10 (14.28%)
	PRES	5	7.1	
Gyral edema	Gyral edema (post_ictal)	4	5.7	4 5.7%
Negative	Negative MRI	3	4.3	3 4.3%
	Total	70	100	70 100%

MRI successfully detected serious COVID complication namely invasive fungal sinusitis (Fig. 6). On the other hand, MRI failed to detect abnormality in 2 cases that revealed normal MRI in spite that one patient was complaining of severe headache and the other patient had prolonged fatigue.

The correlation between neuro-COVID and respiratory COVID MRI was very week; Pearson's R correlation coefficient was approximately + 0.19, Spearman's correlation coefficient, *r*_*s*_, was 0.276, and those were was statistically insignificant (*p* = 0.2) (Table 5).

**Table 5:** Correlation between MRI findings and lung imaging

MRI findings	CT CHEST findings					Total
	CO-RAD I	CO-RAD II	CO-RAD III	CO-RAD IV	CO-RAD V	
WM micro-hemorrhage	0	0	0	1	1	2
Gyral edema (post_ictal)	0	0	1	3	0	4
Subacute infarction	0	0	1	2	1	4
PRES	0	0	0	2	3	5
Vasculitis	1	0	2	3	6	12
ADEM	0	0	1	1	3	5
Hemorrhagic venous infarction	0	0	1	1	1	3
Non Hgic Venous infarction	0	0	0	0	3	3
Lacunar infarction	1	0	1	2	2	6
Hematoma	0	0	5	7	2	14
Acute infarction	0	1	1	4	3	9
Normal	0	0	1	1	1	3
Total	2	1	14	27	26	70

## Discussion

The COVID-19 outbreak has been the subject of considerable attention all over the world due to its high contagious properties. It primarily targets the respiratory epithelium but also has neuro-invasive potential. Indeed, neuropsychiatric manifestations, such as fatigue, headache, psychiatric symptoms, and delirium, are consistently observed in COVID-19. Neuro-COVID-19 is increasingly becoming an accepted term among scientists and clinicians. The neurobiological basis of viral infections pointed to an ongoing neuroinflammatory response to viral antigens and proinflammatory mediators/immune cells [5].

Interdisciplinary teams should be created to implement strategies for treating the wide range of neurological symptoms in Neuro-COVID-19 patients [4].

The prevalent neurological symptoms among individuals with COVID-19 disease should not be under-estimated during the current pandemic outbreak [6].

This study findings demonstrate that MRI imaging in neuro-COVID-19 has a high clinical impact. And pick up the prevalent and frequent neurological symptoms were the headache (80%), anosmia or olfactory dysfunction (62.9%) was the most frequent symptom. Other symptoms of great significance were stroke like manifestation (motor/ sensory impairment and transient ischemic attack in the form of temporary loss of vision), extrapyramidal manifestation (1.4%) was one of the least frequent symptoms. On the other side; anxiety and low mood (17.1%) is the most frequent psychiatric symptom followed by fatigue (14.3%). Depression (7.1%) is the least psychiatric symptom. These results were in concordance to cross-sectional study done by Carcamo Garcia et al. [7] who found that the most common neurological symptoms were headache (72%), hypogeusia or ageusia (41%), hyposmia or anosmia (40%). While they were in discordant to Study conducted by Soltani et al. [8] which indicated that, the prevalence of CNS or mental associated disorders was 50.68%. The most prevalent symptoms were hyposmia/ anosmia/ olfactory dysfunction, while the prevalence of depression and anxiety was 3.52%. Also study done by Ortelli et al. [9] stated that more than half of patients who recover from COVID-19 experience fatigue. The study conducted by Chuang et al. [10] revealed that impaired consciousness was the most common initial neurologic symptom, followed by stroke, unsteady gait, headache, seizure, syncopal event, acute vision changes, and intracranial hemorrhage.

On the opposite side, Goel et al. [11] proved that the most commonly reported COVID neurological complications are cerebrovascular accidents, encephalopathy, encephalitis, meningitis, and Guillain-Barr e syndrome (GBS).

The comorbidity is potentially complicating the course of COVID-19; 62.9% of patients had associated comorbidities, the most common is HTN in 20 (28.6%) patients, DM were 12 patient (17.1%), morbid obesity 8 (11.4%) patient, cardiac disease 6 (8.6%) patients, and lymphoma were 2 (2.9%) patients. Multiple patients had multiple comorbidities especially in SLE and CKD. These results were in agreement to studies done by Khedr et al. [12] and Gusev et al. [13] who stated that hypertension, diabetes mellitus, ischemic heart disease and rheumatic disorders were the most common comorbidities in patients with CNS affection. In contrast to study of Carcamo Garcia et al. [7] who found that majority (42%) had no prior comorbidities.

In the present study, most patients with Neuro-COVID-19 survived (n = 47); a considerable number of patients died (n = 12); and the rest had unclear outcomes (n = 11). This was in agreement to study of Collantes et al. [14] who demonstrated that most patients with Neuro-SARS survived.

In the present study, no statistical significance between severity of Neuro-COVID and respiratory COVID. In contrary to Gusev et al. [13] who found a relationship between the severity of COVID-19 and the severity and frequency of neurological manifestations. Severe neurological disorders are mostly seen in severe cases of COVID-19 and include acute cerebrovascular accidents (aCVA), acute necrotizing encephalopathy, and Guillain–Barre syndrome. The difference may be a result of different methodology as Gusev et al. [13] studies the hospitalized patients and did not include outpatient clinic cases.

The most common MRI findings were infarctions (acute, subacute, large or lacunar) followed by hematoma. Haemorrhage may be microbleeds in the white matter. Abnormalities in the white matter may results from ADEM or PRES. Post ictal edema can be depicted easily. In 2.8%, false negative imaging, MRI failed to pick up any abnormality to explain neurologic deficit. This was in agreement to studies done by Revzin et al. [15] and Ellul et al. [16] who described variety of cerebro-vascular stroke in neuro-COVID and opposite to Poyiadji et al. [17] who described acute necrotizing encephalopathy as post COVID complication.

Due to the urgency of the COVID-19 pandemic, this study had some limitations. First, it was a single institutional study. Second, as Neuro-COVID patients had varying degrees of disease severity, from light headache to critically ill patients and MRI was performed commonly in severely ill patients, patient selection bias is possible. Third, the lack of a control group limits our findings to Neuro-COVID-19. Forth, the lack of histopathologic data is also a limitation. Finally, the limited availability of long-term clinical outcomes.

These limitations necessitate international longitudinal studies for a more detailed analysis of co-morbidities and to determine the long-term neurological sequelae of COVID-19 during the acute and post-infectious period [18].

## Conclusion

During the current pandemic, neurologists should be aware of the possibility of Neuro-COVID 19 infection in patients with neurological damage in the emergency department to avoid misdiagnosis or the spread of infection. Hospitalized patients with COVID-19 infection may show symptoms of neurological damage, such as cerebrovascular disease, and demyelinating lesions. However, no statistical significance between neuroimaging and lung imaging in COVID-19 patients.

## Data Availability

The datasets used and/or analysed during the current study are available from the corresponding author on reasonable request.

## Abbreviations

CoVs: Coronaviruses
COVID 19: Novel coronavirus
WHO: World Health Organization
SD: Standard deviation
HTN: Hypertension
DM: Diabetes mellites
SLE: Systemic lupus erythematosus
CKD: Chronic kidney disease
PRES: Posterior reversible encephalopathy
ADEM: Acute disseminated encephalomyelitis
